# Mr. Gladstone on the Medical Profession

**Published:** 1894-05

**Authors:** 


### Editorial.

### MR. GLADSTONE ON THE MEDICAL PROFESSION.

A recent meeting, held in London in memory of the late Sir Andrew Clark, deserves to receive particular attention of medical men, for it was indirectly a testimonial to the greatness of our profession, as well as to one of its most distinguished and beloved members. The meeting was presided over by the Duke of Cambridge, and was attended by Mr. Gladstone, whose remarks were of great and special interest.

Mr. Gladstone, in offering a resolution that a memorial be established which shall perpetuate the name and work of Sir Andrew Clark, said, in part:

"For my own part, sir, it has been with some small effort that I have come here to bear my humble part in the proceedings of the day. To have been absent from them, to have missed the opportunity of bearing testimony to the noble life of Sir Andrew Clark, would have been to me a standing cause of grief and mortification.

"The profession itself is one with regard to which it is impossible, I think, not to be conscious that, great as has been its position in our generation and some generations previous, it is a position continually advancing and continually rising. In truth, your Royal Highness, if we go back some distance, we find that the other learned professions, as they are sometimes called, undoubtedly had a start of the medical profession. I think, as much as four or five hundred years ago, in the first beginning of the commerce of this country, the lawyers in scores and hundreds sprang up, I might almost say like mushrooms from the ground, to dispose of the disputes which necessarily arise wherever property has been brought into existence. At that time medical men had hardly been heard of. But the position of the medical profession to-day is becoming one of vital and commanding interest to the whole of society, and I anticipate that that interest must continue. While wealth increases, while inventions and discoveries increase, wants will increase and enjoyments will increase; and, in connection with those wants and enjoyments there will, I fear, your Royal Highness, be a corresponding increase of infirmity and disease, and the medical profession braces itself to grapple with the situation which has been created, and continually advances in knowledge and in competence. My own life has been long enough to enable me to witness, and in some degree to measure, the change that has taken place. I have had the honor of knowing many eminent and distinguished men in the profession during the last three-score years, and I have seen also great change in capacity, in attainment, and in competency to deal with the difficult, the almost insoluble, problems that are continually presenting themselves to the mind of the medical man. Well, Sir, it appears to me that it was eminently desirable that, in a time like this, a man like Sir Andrew Clark should rise to the head of the profession—for, after all, we require something more than knowledge, something more than skill; we require great devotion to the cause of the profession; and that devotion was never, I think, exemplified in a more remarkable manner than in the career of Sir Andrew Clark. When he had been spending his well-earned vacation at a residence he temporarily occupied in Scotland, and when the few weeks he had allowed himself for retirement were nearly exhausted, a friend came, and, supposing he was paying him a compliment, said, 'I condole with you, Sir Andrew Clark, on the close of your vacation.' Sir Andrew turned and said, 'Sir, I love my profession.' He did love the profession. His whole heart and soul were in the profession. He loved it not only with sincerity and cordiality, but with a chivalrous devotion. We need not say the age of chivalry is altogether past, so long as we have among us —and I trust we may still have among us—men of the type of Sir Andrew Clark."

Such words as these, coming from a person of a mind so broad and experience so vast as that of Mr. Gladstone, give encouragement and renewed zest to all physicians who, like Sir Andrew Clark, love their profession and believe in its present greatness and future advancement.—*Medical Record.*

